# The *STK35* locus contributes to normal gametogenesis and encodes a lncRNA responsive to oxidative stress

**DOI:** 10.1242/bio.032631

**Published:** 2018-07-03

**Authors:** Yoichi Miyamoto, Penny A. F. Whiley, Hoey Y. Goh, Chin Wong, Gavin Higgins, Taro Tachibana, Paul G. McMenamin, Lynne Mayne, Kate L. Loveland

**Affiliations:** 1 Department of Biochemistry and Molecular Biology, School of Biological Sciences, Monash University, Wellington Road, Clayton, VIC 3800, Australia; 2 Laboratory of Nuclear Transport Dynamics, National Institutes of Biomedical Innovation, Health and Nutrition, 7-6-8 Saito-Asagi, Ibaraki, Osaka 567-0085, Japan; 3 Centre for Reproductive Health, Hudson Institute of Medical Research, 27-31 Wright Street, Clayton, VIC 3168, Australia; 4 Department of Bioengineering, Graduate School of Engineering, Osaka City University, Osaka 558-8585, Japan; 5 Department of Anatomy and Developmental Biology, School of Biological Sciences, Monash Medical Centre, 246 Clayton Road, Clayton, VIC 3168, Australia; 6 Department of Molecular and Translational Sciences, School of Clinical Sciences, Monash Medical Centre, 246 Clayton Road, Clayton, VIC 3168, Australia

**Keywords:** Long non-coding RNA, Spermatogenesis, Oogenesis, Eye development, Hydrogen peroxide

## Abstract

Serine/threonine kinase 35 (STK35) is a recently identified human kinase with an autophosphorylation function, linked functionally to actin stress fibers, cell cycle progression and survival. *STK35* has previously been shown to be highly expressed in human testis, and we demonstrated its regulation by nuclear-localized importin α2 in HeLa cells. The present study identifies progressive expression from the *STK35* locus of two coding mRNA isoforms and one long non-coding RNA (lncRNA) in mouse testis during spermatogenesis, indicating their tightly controlled synthesis. Additionally, lncRNA transcripts are increased by exposure to oxidative stress in mouse GC-1 germ cell line. *STK35* knockout (KO) mice lacking all three RNAs are born at sub-Mendelian frequency, and adults manifest both male and female germline deficiency. KO males exhibit no or partial spermatogenesis in most testis tubule cross-sections; KO ovaries are smaller and contain fewer follicles. Eyes of KO mice display phenotypes ranging from gross deformity to mild goniodysgenesis or iridocorneal angle malformation, to overtly normal. These findings demonstrate the tight regulation of transcription from the *STK35* locus and its central importance to fertility, eye development and cell responses to oxidative stress.

## Abstract (precis)

**Summary:** Transcripts from the STK35 locus impact on male and female fertility and on eye development. A lncRNA (*Stk35os1*) transcript from this locus is upregulated by oxidative stress.

## INTRODUCTION

There is accumulating evidence identifying products of the *serine/threonine kinase 35* (*STK35*) gene as suitable targets for diagnosis or intervention in cardiac pathologies (Lamore et al., 2017; Yang et al., 2014), colorectal and other cancers (Capra et al., 2006) and malaria (Prudêncio et al., 2008), hence investigations of its physiological roles are of great interest. STK35 was originally identified as a binding partner of the PDZ-LIM protein, CLP-36, and named Clik1, for CLP-36 interacting kinase 1. It was shown to have autocatalytic activity and be highly expressed in human testis (Vallenius and Makela, 2002). Subsequent discovery of an alternate upstream transcription start site (TSS) resulted in the identification of an N-terminus long form, termed STK35L1 (Goyal et al., 2009). Knockdown of *STK35L1* by siRNA in Human Umbilical Vein Endothelial cells (HUVECs) accelerated cell cycle progression from G1 into S phase, and was accompanied by transcriptional inhibition of key cell cycle regulatory genes and of DNA damage and stress response genes, such as *CDKN2A* and *GADD45A* (Goyal et al., 2011)*.* Thus, STK35L1 has been implicated in both maintenance of normal cell cycle progression and in controlling the expression of genes involved in DNA damage and cellular stress responses.

We previously reported that the *STK35* mRNA level was uniquely upregulated in HeLa cells exposed to hydrogen peroxide (H_2_O_2_) through the actions of importin αs, which are classical nuclear localization signal (NLS) receptors that mediate cargo protein transport into the nucleus (Yasuda et al., 2012). When cultured cells are exposed to cellular stresses including oxidative stress, importin α protein rapidly accumulates in the nucleus, and selectively regulates several genes, including *STK35* (Yasuda et al., 2012). *STK35* depletion by siRNA protected cells from dying, whereas ectopic over-expression of STK35 enhanced non-apoptotic cell death under oxidative stress, hence we proposed that STK35 is a stress-responsive molecule involved in cell fate determination (Miyamoto et al., 2012; Yasuda et al., 2012).

Several databases, including NCBI, indicate *STK35* RNA levels are high in the mouse testis and ovary relative to other tissues. In particular, FANTOM5 shows *STK35* levels are highest in testis, ovary and eye, suggesting that *STK35* may make an important contribution to cellular functions in these organs (Lizio et al., 2015).

Here we provide an improved understanding of the *STK35* genetic locus and the *in vivo* functional importance of its transcripts from analyses of a knockout (KO) mouse. The mouse genome encodes two protein-coding messenger RNA isoforms at the *STK35* gene locus on the sense strand in addition to a lncRNA on the antisense strand. The synthesis of these transcripts appears to be coordinated during the developmental progression of spermatogenesis. Our newly developed *STK35* KO mouse, in which both coding and non-coding RNAs are deleted, revealed striking impairment of germline development in both testis and ovary, causing subfertility in addition to eye phenotypes. The significant upregulation of the lncRNA in response to hydrogen peroxide exposure in germline cells suggest that differential transcription from the *STK35* locus mediates processes essential for normal growth within dynamic environments that include states of changing oxygen tension.

## RESULTS

### *STK35* transcript survey in mouse

**Fig. 1.**
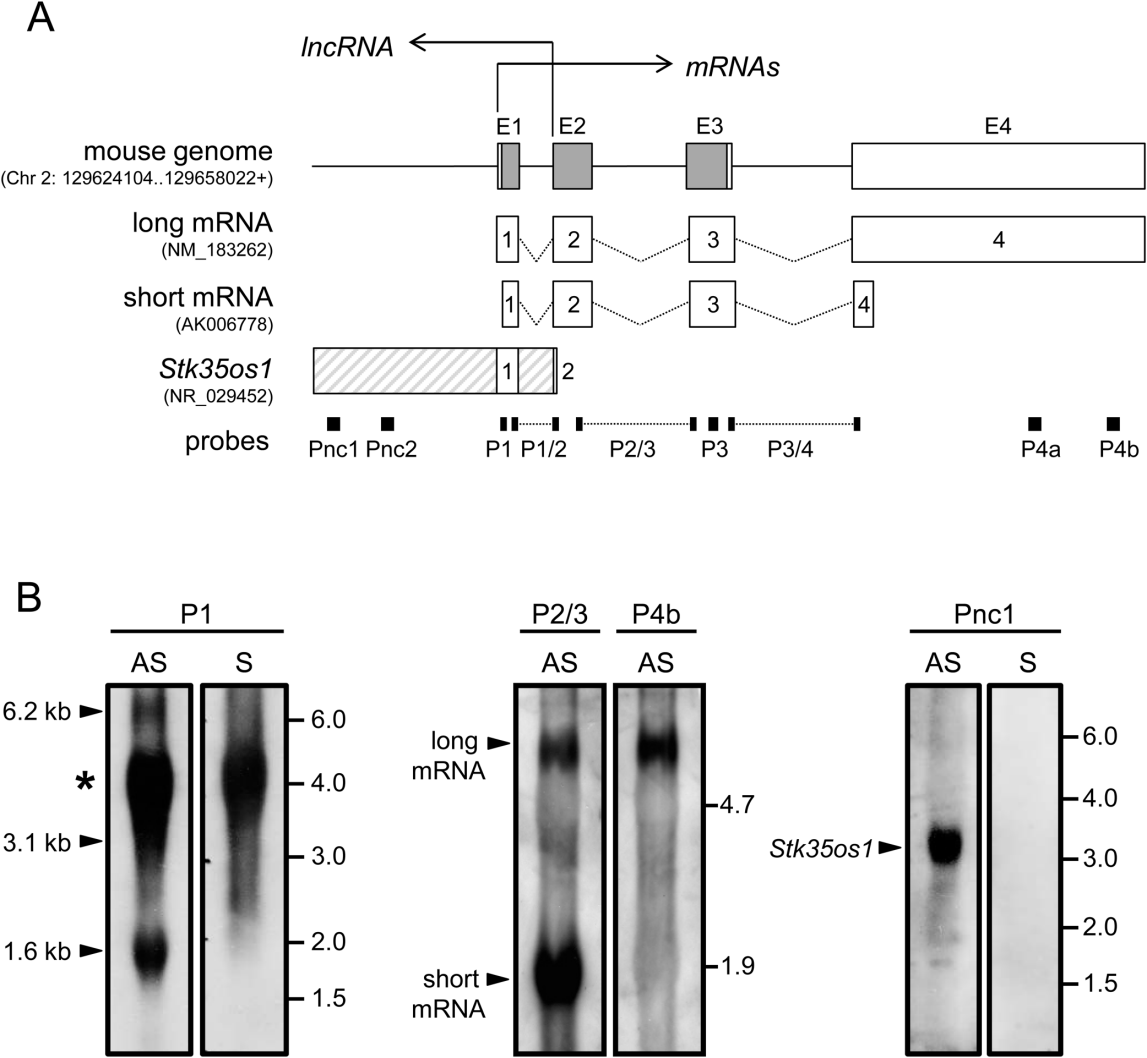
***STK35* transcript survey in the mouse testis.** (A) Schematic representation of *STK35* gene locus and transcripts expressed in adult mouse testis. Gene locus co-ordinates obtained from FANTOM5 (fantom.gsc.riken.jp/5/). The *STK35* gene consists of four exons (E1-E4) on chromosome 2. The protein coding region (highlighted in grey) is located in exons 1–3, and exon 4 contains the 3′-UTR. Two coding transcripts, long mRNA (NM_183262) and short mRNA (AK006778) are 6.1 kb and 1.6 kb, respectively. One lncRNA, ‘*Stk35os1*’, 3.1 kb in size, is positioned in a 5′ head-to-head orientation to *STK35*. Probes (P1-P4b, Pnc1 and Pnc2) are represented by black bars and their approximate location indicated. (B) Northern blot analysis using P1 AS (antisense) and S (sense) probes in adult mouse testis. A strong background signal was detected at 4.0 kb as indicated by the asterisk. Probe P2/3 AS detects *STK35* long and short transcripts, P4b AS detects *STK35* long transcript, and probe Pnc1 AS, but not S, detects *Stk35os1* lncRNA. Molecular size markers (kb) are indicated on the right-hand side of each panel.

We first sought to identify the *STK35* transcripts present in adult mouse testis. The murine *STK35* gene is on chromosome 2 and consists of 4 exons. The protein coding region is located in exons 1–3, similar to the human *STK35* gene (Goyal et al., 2009), while exon 4 (E4) contains the 3′-UTR and two polyadenylation signals (Fig. S1A). Four mRNA variants are annotated in UniGene (NCBI: http://www.ncbi.nlm.nih.gov/unigene, UGID:1999943-UniGene Mm.389329), NM_183262, NM_001038635, BC047277 and AK006778, of which three appear to possess poly(A) tails (Fig. S1B). In addition, this locus codes for a validated lncRNA designated in UniGene (UGID:1104591-UniGene Mm.305555) as 4932416H05Rik (NR_029452.1), which corresponds to a single exon transcript in a 5′ head-to-head orientation to the *STK35* coding gene (Fig. 1A). This lncRNA, renamed by us, according to mouse nomenclature guidelines (http://www.informatics.jax.org/mgihome/nomen/gene.shtml) as *serine/threonine kinase 35 opposite strand 1* (*Stk35os1*), initiates within the second exon of *STK35* and overlaps with the first 362 nucleotides of the coding RNA.

Northern blotting was performed to identify RNA variants present in adult mouse testis. Probes were designed to distinguish between *STK35* coding and non-coding transcripts, however sequence similarity between coding transcripts prevented the design of primers capable of detecting the short mRNA transcript alone. Probe names indicate the exonic sequences they include. Probes P1 and P1/2 were designed to recognize both *STK35* mRNAs and *Stk35os1*; probe P1 detected three bands at 6.2 kb, 3.1 kb and 1.6 kb (Fig. 1B, first panel). P1/2 showed the same bands as probe P1 (data not shown). Probe P2/3, designed to detect both *STK35* mRNAs but not the lncRNA, detected bands at 6.2 kb and 1.6 kb, whilst probe P4b, corresponding to the 3′ end of exon 4 present in the longer mRNA, detected only the long (6.2 kb) mRNA band (Fig. 1B, second panel). Probes P3 and P3/4 showed the same bands as probe P2/3, and P4a detected the same 6.2 kb band as probe P4b (data not shown). Probe Pnc1 (probe non-coding 1), designed to detect *Stk35os1*, recognized a single band at 3.1 kb (Fig. 1B, third panel). In summary, these northern blots identified two prominent *STK35* mRNAs [a 6.2 kb ‘long mRNA’ (NM_183262) and a 1.6 kb ‘short mRNA’ (AK006778)], and one 3.1 kb lncRNA (*Stk35os1*) in adult mouse testis (Fig. 1A). The NM_001038635 (5.4 kb) and BC047277 (1.4 kb) splice variants were not detected.

### Cellular expression profiles of *STK35* isoforms and *Stk35os1* in mouse testis

**Fig. 2.**
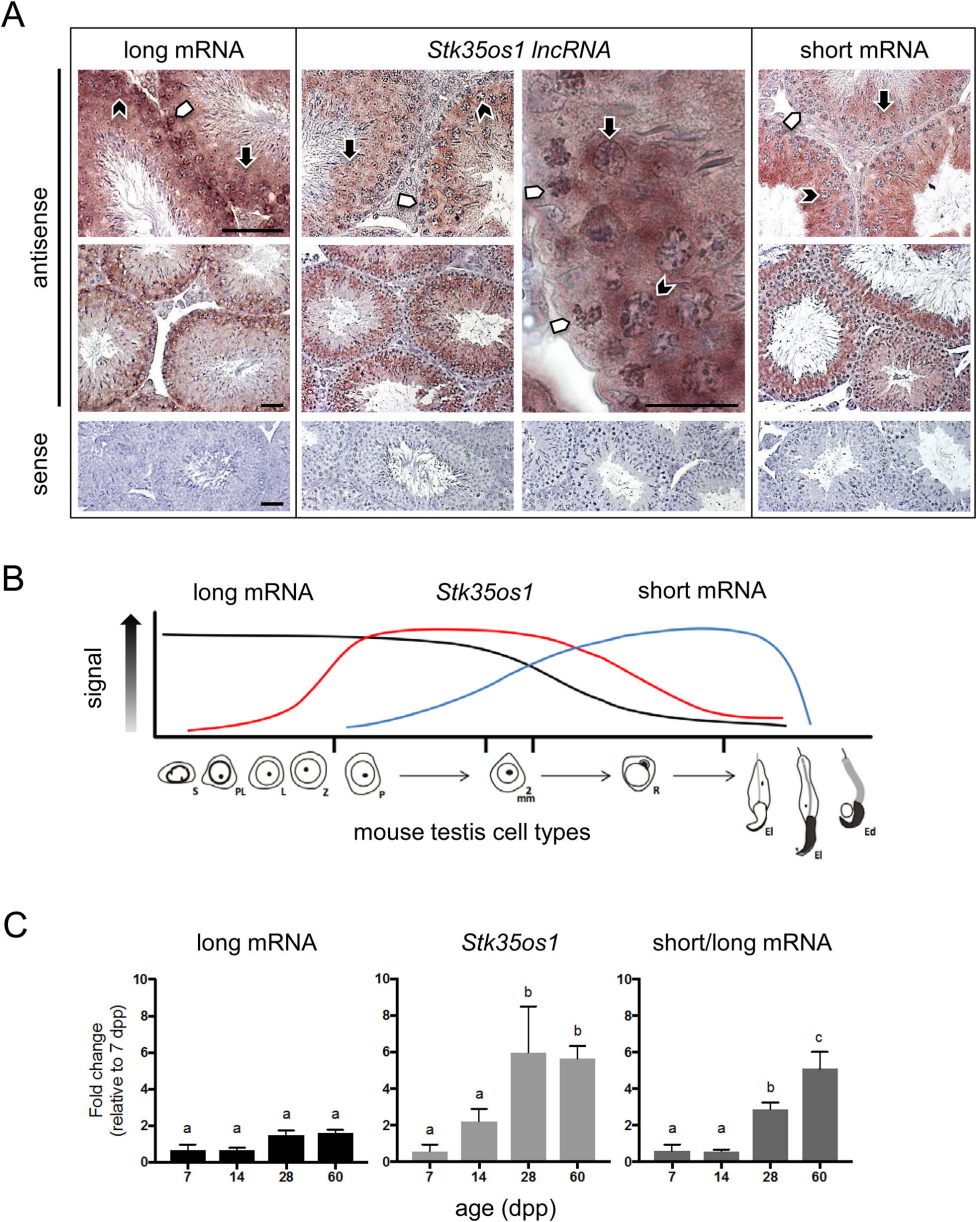
***STK35* RNAs are expressed in distinct yet overlapping cell populations in adult mouse testis.** (A) *In situ* hybridization of adult mouse testis with P4b (long mRNA), Pnc1 (*Stk35os1*) and P2/3 (short mRNA), antisense and sense probes. Spermatogonia, white arrowheads; pachytene spermatocytes, black chevron; round spermatids, black arrow. Scale bars: 50 µm and are representative of all images on that line, unless otherwise indicated. (B) Diagram summarizing the expression of *STK35* long mRNA, *Stk35os1* lncRNA and *STK35* short mRNA in the major cell types of the mouse seminiferous epithelium: S, spermatogonia; PL, preleptotene; L, leptotene; Z, zygotene; P, pachytene spermatocyte; mm, meiotic divisions; R, round spermatids; El, elongating spermatids; Ed, elongated spermatids. (C) Transcript levels of *STK35* long (P4b), *Stk35os1* (Pnc1) and short/long (P2/3) mRNAs were analyzed by qRT-PCR on 7, 14, 28 and 60 dpp Swiss Asmu mouse testes (*n*=3/age). Data are presented as fold-change compared to 7 dpp (mean±s.d.). Significance determined using two-way ANOVA with Tukey's multiple comparisons test. Lowercase letters a, b and c indicate values within each graph that are significantly different (*P*<0.01).

To identify cells expressing the *STK35* isoforms and *Stk35os1* lncRNA in adult mouse testis, we employed probes P4b, Pnc1 and P2/3 for *in situ* hybridization analysis. The most predominant signals for each were in spermatogenic cells and are described here. Probe P4b, which detects only the long mRNA, yielded the strongest signal in the least mature germ cell types, showing an intense signal in spermatogonia and early spermatocytes, and lesser signals in pachytene spermatocytes and round spermatids (Fig. 2A). The *Stk35os1* lncRNA specific probe, Pnc1, was detected predominantly in pachytene spermatocytes and round spermatids (Fig. 2A). Because probe P2/3 can bind to both long and short *STK35* transcripts, we expected to see a signal with this probe in spermatogonia, as seen with probe P4b. However, during *in situ* hybridization development, the first observed signal was in more mature germ cells, with the most intense signal in round and early elongated spermatids (Fig. 2A). Continued development did result in a signal in spermatogonia, as predicted. Based on the initial detection of haploid germ cells with P2/3, we consider that this probe will predominantly detect the short transcript by *in situ* hybridization. This approach provided evidence that the three transcripts are sequentially expressed during spermatogenesis in the adult mouse testis (Fig. 2B).

Quantitative Real Time-PCR (qRT-PCR) was used to assess whether expression of each transcript during the first wave of spermatogenesis demonstrated a developmentally regulated expression profile. Whole testis RNA was examined at progressive postpartum ages when each of the major spermatogenic cell populations first emerge and then become numerically dominant. The long mRNA transcript levels (P4b primers) in whole mouse testis were not significantly altered between samples aged from 7 dpp, when spermatogonia are the only germ cell type present, through to adulthood (Fig. 2C). This correlates with *in situ* hybridization data illustrating its predominant detection in spermatogonia and early spermatocytes (Fig. 2B). *Stk35os1* (Pnc2 primers) increased when pachytene spermatocytes first emerged by 14 dpp, and was significantly higher at 28 dpp, when spermatocytes are the predominant testicular cell type (Fig. 2C). In contrast, the mRNAs amplified by the P2/3 primers were relatively low at 7 and 14 dpp, became significantly higher at 28 dpp as round spermatids emerged, and then significantly increased further to a peak value at 60 dpp when haploid germ cells (early and late spermatids) are the majority cell type in the testis (Fig. 2C). These outcomes further demonstrate the distinct but overlapping expression profiles of the *STK35* mRNAs and *Stk35os1* lncRNA, and they indicate that each transcript is differentially regulated to serve distinct functions during spermatogenesis.

### *Stk35os1* transcript levels increase in response to oxidative stress

**Fig. 3.**
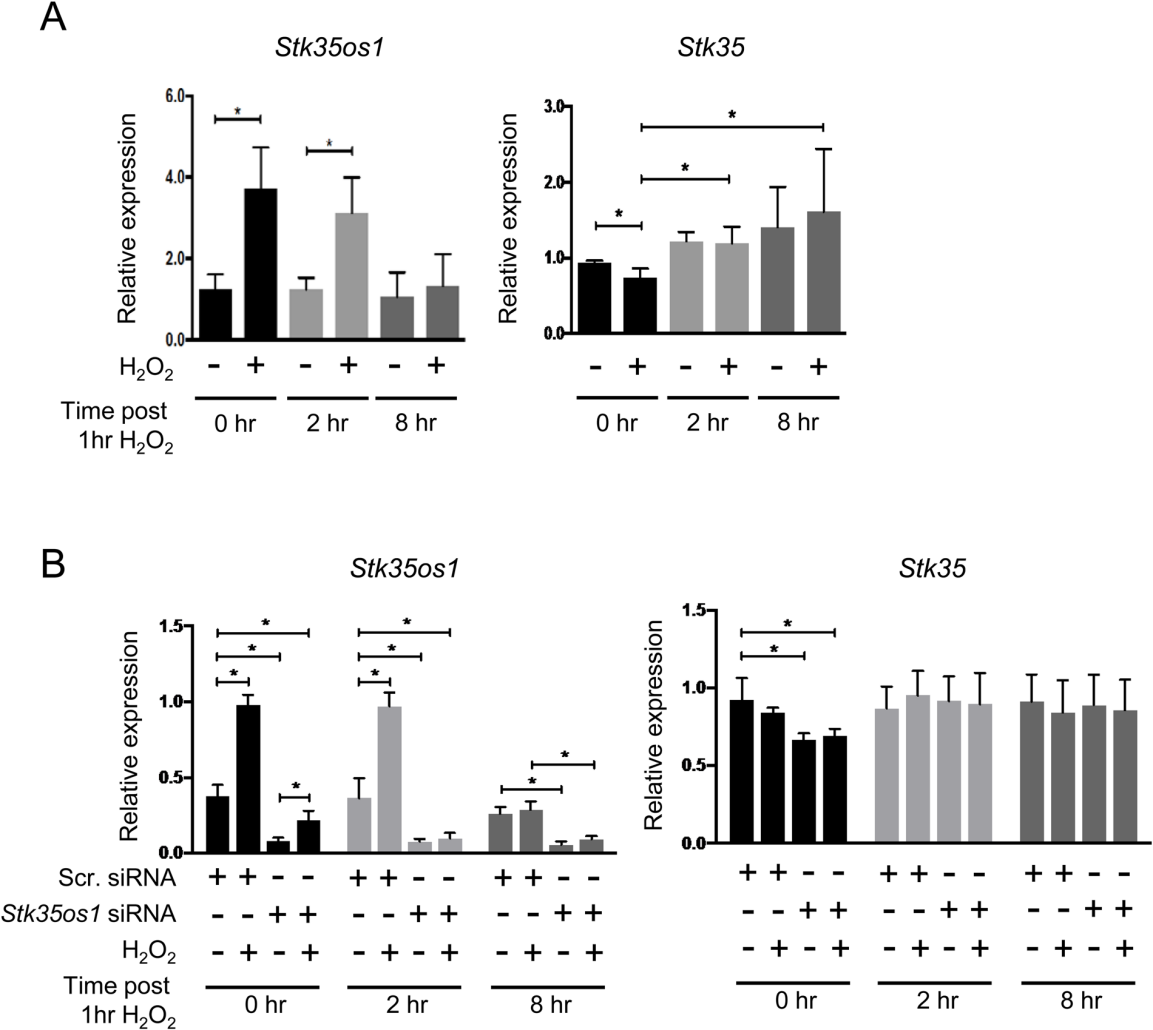
***Stk35os1* lncRNA is upregulated in response to oxidative stress.** (A) Quantitative RT-PCR analysis for *Stk35os1* (Pnc2) and *STK35* (P2/3) transcripts in GC-1 cells ±1 mM H_2_O_2_ for 1 h, collected immediately after treatment at 0 h, 2 h and 8 h post-treatment. (B) *Stk35os1* (Pnc2) and *STK35* (P2/3) transcripts are shown following transfection with scrambled (Scr.) siRNA, *Stk35os1* siRNA, in the absence and presence of 1 mM H_2_O_2_ at 0 h, 2 h and 8 h. All graphs show relative expression for *n*=4 independent experiments (mean±s.d.). Statistical significance was determined using unpaired Mann–Whitney *t*-test, where *P<0.05.

We had previously described altered *STK35* mRNA levels in HeLa cells exposed to H_2_O_2_ (Yasuda et al., 2012). Our *in situ* hybridization data identifying developmentally regulated synthesis of three transcripts from the *STK35* locus in testicular germ cells prompted us to examine the relationship between *STK35* allele transcription and oxidative stress in the male germline. To do this, we used the mouse GC-1 cell line which was originally derived from spermatogonia (Hofmann et al., 1992) and has been used extensively to study proliferative germline cells. GC-1 cells were exposed to H_2_O_2_ for 1 h before media was replaced with normal culture medium; samples were collected immediately following treatment (0 h), and at 2 h and 8 h later. *Stk35os1* (Pnc2) exhibited a substantial and significant upregulation at 0 h and 2 h (two- to threefold increase) and returned to untreated levels by 8 h (Fig. 3A, left graph). Initial experiments in the GC-1 cell line used primer sets P2/3 and P4b to detect the short/long and long *STK35* transcripts, respectively. Since both primers sets generated identical results (data not shown), further experiments were performed using primers P2/3, which detected both *STK35* isoforms. *STK35* coding transcripts (P2/3) exhibited a small but significant decrease at 0 h, however these showed an overall increase in H_2_O_2_-exposed samples over the 8 h examined, similar to the change reported in HeLa cells (Yasuda et al., 2012) (Fig. 3A, right graph). Since lncRNAs may directly regulate neighboring gene transcription (Carrieri et al., 2012), we next tested whether lowering the *Stk35os1* lncRNA level would change the impact of oxidative stress on *STK35* transcription in GC-1 cells. Cells transfected with scrambled siRNA (scr.) or siRNA targeted to *Stk35os1* were treated with or without H_2_O_2_ for 1 h, then samples were collected immediately (0 h), or 2 and 8 h later. In scr. siRNA-treated controls, *Stk35os1* transcript levels were increased by H_2_O_2_ at 0 and 2 h post-treatment, as previously documented (Fig. 3A). Although present at a lower level in all siRNA treated samples (<70–75% compared with scr. siRNA control; Fig. 3B left graph), *Stk35os1* was elevated 2.2-fold immediately after 1 h of H_2_O_2_ exposure (0 h time point), with no significant difference recorded at the 2 h and 8 h time points (Fig. 3B left graph). *STK35* levels were slightly, but significantly, decreased in *Stk35os1* siRNA samples, in both H_2_O_2_-treatment and control groups at 0 h (Fig. 3C right graph), but not at subsequent time points. These data demonstrate that induction of *Stk35os1* lncRNA transcripts in response to stress is robust and indicate that any reciprocal relationship between *Stk35os1* and *STK35* mRNA levels following exposure to oxidative stress is likely to be minimal and transient.

### Targeted disruption of the *STK35* gene

**Fig. 4.**
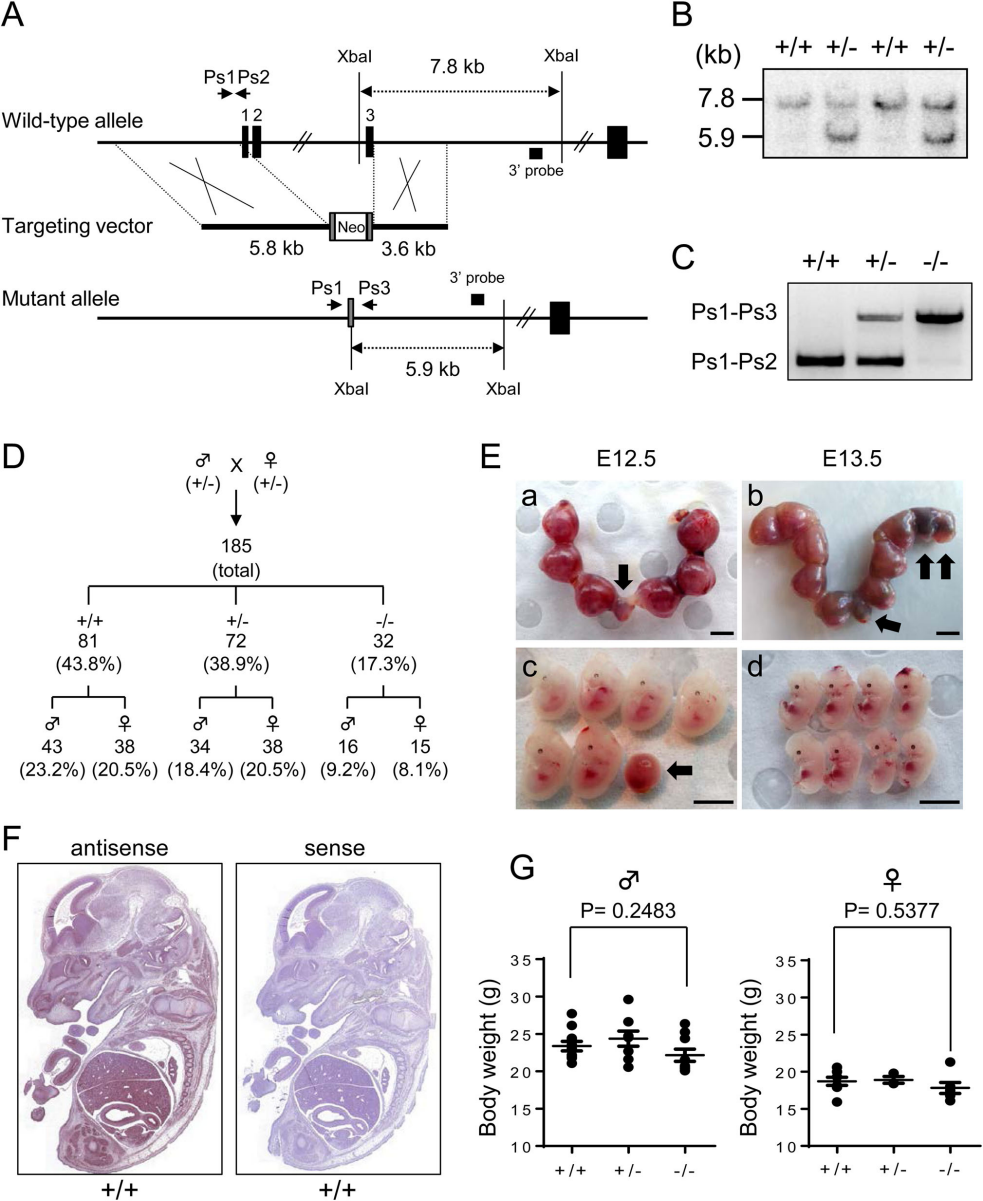
**Targeted disruption of *STK35* transcripts in mouse.** (A) Gene targeting strategy for *STK35* KO mice. After homologous recombination, exons 1, 2 and 3 were replaced by a FRT-flanked neomycin (Neo) cassette. PCR primers for genotyping are shown as Ps1, Ps2 and Ps3. (B) Southern blot analysis of *Xba*I-digested DNA isolated from ES clones. Recombination of the 3′-ends detected as a change of a 7.8 kb WT *Xba*I fragment to a novel 5.9 kb fragment using the indicated 3′ probe. (C) PCR analysis using genomic DNA of the indicated genotypes. (D) Genetic analysis of pups generated from heterozygous parents. A total 185 mice were counted, with the sex ratio of each genotype recorded. (E) Uteri isolated from *STK35* Het mice at embryonic 12.5 day (E12.5; a,c) and E13.5 (b,d), in which the exhibited embryos (c,d) were obtained from uteri (a,b), respectively. Dead embryos are indicated by black arrows. Scale bars: 5 mm. (F) *In situ* hybridization analysis of E13.5 whole WT (+/+) embryos using probe P2/3. (G) Body weight of male and female mice (>40 dpp) for each genotype (*n*=8 each).

To explore *STK35* gene product function *in vivo*, we generated a KO mouse in which a targeting construct was designed to delete exons 1, 2 and 3, spanning approximately 10 kbp (Fig. 4A). Southern blot analysis at both the 5′ and 3′ ends of the homologous recombination event confirmed targeted ES clones, with the 3′ end recombination detected as the change of a 7.8 kb wild-type (WT) *Xba*I fragment to a novel 5.9 kb recombinant fragment (Fig. 4B). The homozygous *STK35* mutation mice (−/−; KO) were obtained from matings between heterozygous (+/−; Het) F1 and F2 mice, and the line was subsequently maintained by heterozygote breeding onto a C57Bl6/J background for a minimum of six generations. Genotyping was performed using primers Ps1 and Ps2 to amplify a product of 345 bp from the WT allele and primers Ps1 and Ps3 to amplify a 612 bp product from the targeted flp'd allele (Table S1; Fig. 4C).

F1 heterozygous female and male matings produced homozygous KO mice in a non-Mendelian ratio (1:0.9:0.4), but the sex ratio was unaffected (Fig. 4D). Several embryos aged from E12.5 to E13.5 showed developmental defects and lethality (Fig. 4E). *In situ* hybridization analysis revealed that *STK35* transcripts are extensively expressed in mice at E13.5 (Fig. 4F), indicating that loss of heterozygous and homozygous fetuses could result from *STK35* deficiencies in embryogenesis. While gross physical examination demonstrated that adult *STK35* KO mice generally have a lower body mass compared to their WT siblings, this difference was not statistically significant (Fig. 4G).

### *STK35* KO mouse testicular defects

**Fig. 5.**
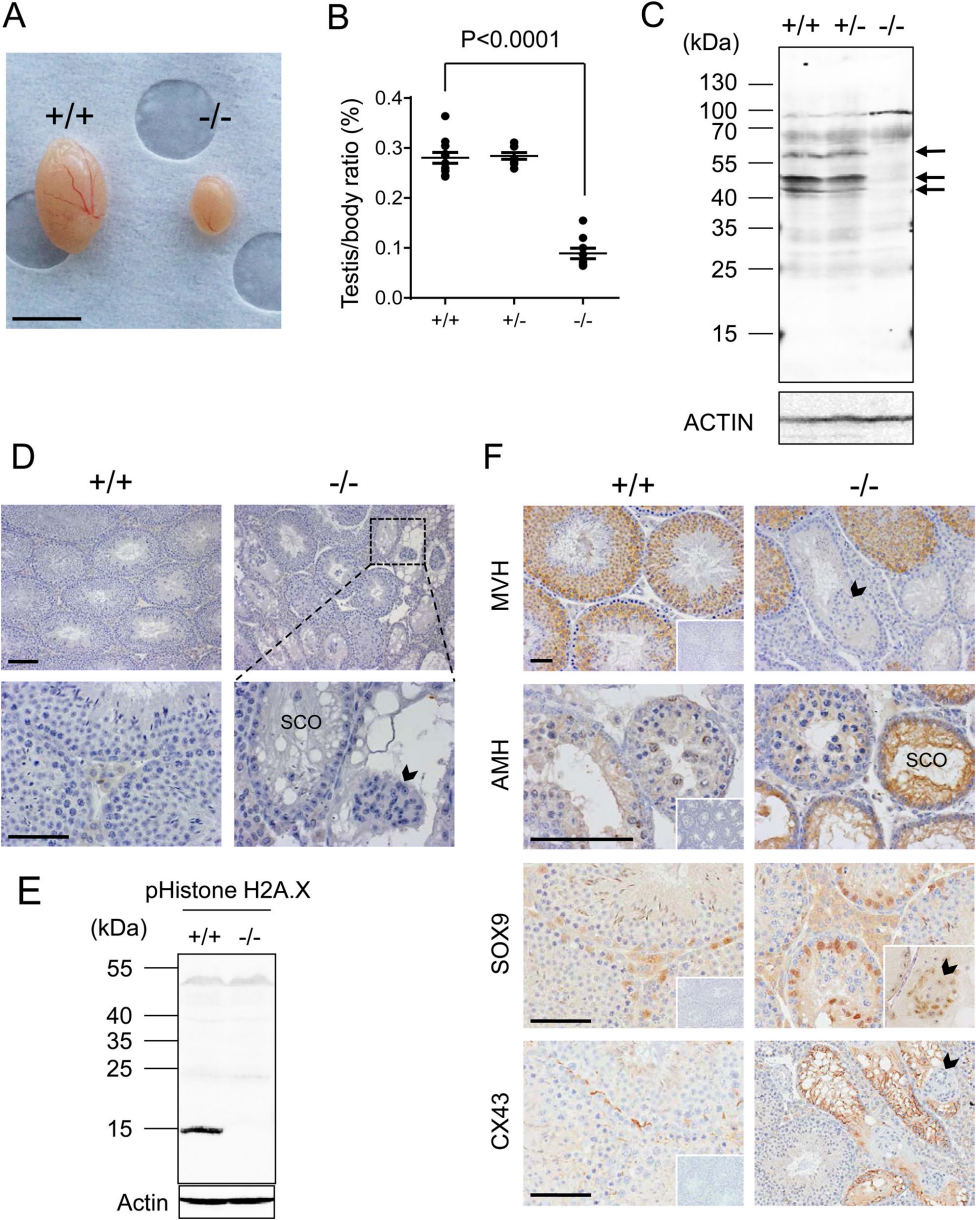
**Testis defects in *STK35* KO mice.** (A) Representative image of *STK35* WT (+/+) and KO (−/−) testes. Scale bars: 5 mm. (B) Graph showing testis/body weight ratio of KO mice was significantly lower than for WT or Het mice. (C) Western blot confirming STK35 proteins are absent in KO mouse testis (arrows). (D) Histological comparison of *STK35* WT and KO tissues revealed severe disruption in spermatogenesis in KO testes. Sertoli cell only (SCO) tubule and Sertoli cell cluster indicated by arrowhead. (E) Western blot analysis of phospho-Histone H2A.X on WT and KO mice testes. (F) Immunohistochemical analysis of *STK35* WT and KO testis sections with MVH, AMH, SOX9 and CX43 antibodies. Cellular localization of MVH, SOX9 and CX43 proteins are shown in adult mouse testis, and AMH in 21 dpp mouse testis. A representative negative control image for each antibody is shown on WT (inset). Sertoli cell only tubules and Sertoli cell clusters indicated by SCO and arrowheads respectively. Scale bars: 50 µm for D,F and are representative of corresponding KO image.

An exhaustive phenotype analysis by the Australian Phenomics Network (Melbourne) comparing male littermates from the *STK35* colony provided the first indication that the testis was selectively affected by the absence of *STK35*. Testis mass and testis/body-weight ratio were significantly lower in KO mice compared to WT and Het littermates (Fig. 5A,B). Mating with WT females resulted in smaller litter sizes from KO males compared to from either WT or Het males (WT, 7.8±1.3; Het, 6.1±0.5; KO, 4.0±0.6). To validate the absence of a signal in *STK35* mouse knockout tissue, both commercial (*n*=2) and in-house (*n*=2) antibodies were trialed using several immunohistological methods; none gave a distinct signal in testis sections of WT mice, so only western blots using whole testis samples could be used. Our in-house antibody (Fig. S2) detected three prominent bands in adult testes by western blot analysis, at 55 kDa, 50 kDa and 45 kDa, which were not detected in the KO testis (Fig. 5C). The 55 kDa band corresponds to the size of the full-length STK35L1 protein (corresponding to NM_183262), and we suggest the two smaller bands may either be the shorter STK35/CLIK1 protein with its phosphorylated isoform, or degraded products originating from STK35L1.

Haematoxylin staining revealed highly heterogeneous disruption to spermatogenesis, as demonstrated by counting the proportion of tubule cross-sections with abnormalities; this ranged from 65% to 100% and 13% to 81% of total tubules in testes from different KO individuals at 21 dpp and in adulthood (60–90 dpp), respectively. The more severe phenotypes observed in the KO testes featured extensive germinal epithelium vacuolization, Sertoli cell sloughing into the tubule lumen (indicated by black arrowhead, confirmed by staining with the Sertoli cell marker, SOX9, Fig. 5F) and areas lacking germ cells [Sertoli-cell only (SCO), Fig. 5D,F]. The histone H2A.X protein is present in most germ cells (spermatogonia to round spermatids), and its phosphorylated isoform is detected in the germline during normal chromatin remodeling events such as meiosis, in the absence of DNA double strand breaks induced by ionizing radiation (Hamer et al., 2004). While WT testes contained abundant phospho-Histone H2A.X, readily detected by western blot, its absence in KO testes provided further evidence of post-mitotic germ cell loss (Fig. 5E). Immunohistochemical detection of mouse vasa homologue (MVH) protein revealed tubule cross sections with normal spermatogenesis adjacent to SCO regions (Fig. 5F). Sertoli cells in tubules lacking germ cells expressed anti-Müllerian hormone [AMH, also known as Müllerian-inhibiting substance (MIS)], an indicator of Sertoli cell immaturity or reduced capacity to support germ cell development (Sharpe et al., 2003). Staining with SOX9 also indicated some Sertoli cell nuclei were abnormally located, away from the tubule perimeter (Fig. 5F). Germ cell-deficient tubules displayed intense Connexin 43 (CX43) staining, indicating loss of the blood-testis barrier integrity normally formed between adjacent Sertoli cells and essential to sustain post-mitotic spermatogenesis. These findings indicate that the absence of *STK35* results in male germ cell loss and Sertoli cell dysfunction, and decreased male fertility.

### Abnormal ovary development in *STK35* mutants

**Fig. 6.**
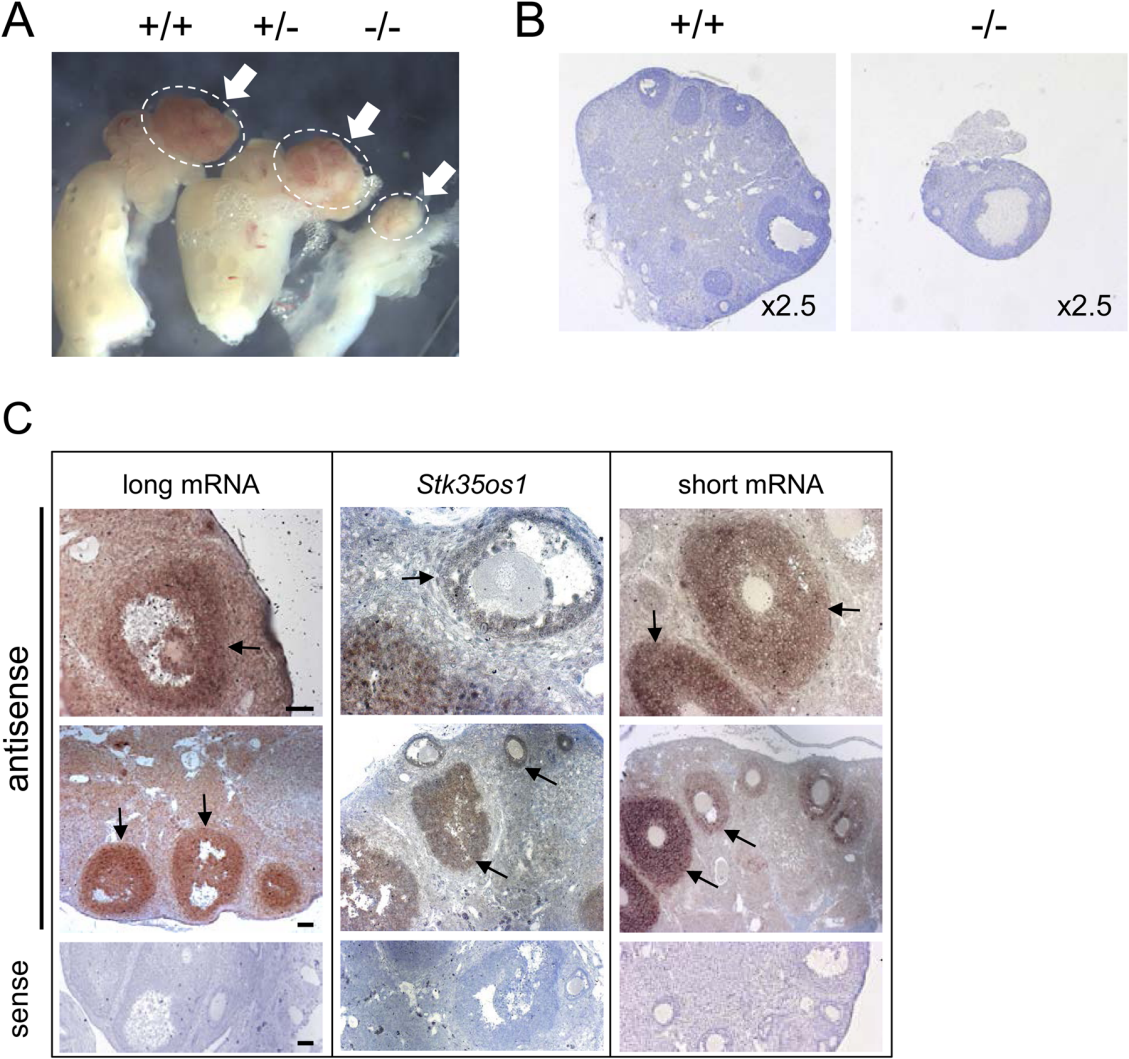
***STK35* KO mice show ovary defects.** (A) Representative image of *STK35* WT (+/+), Het (+/−) and KO (−/−) ovary (circled by white dotted line). (B) Histological comparison of *STK35* WT and KO ovary revealed reduced organ size and follicle number in the KO ovary. (C) *In situ* hybridization in adult mouse ovary with probes P4b (long mRNA), Pnc1 (*Stk35os1*) and P2/3 (short mRNA) shows all three transcripts are predominantly expressed in the granulosa cells (black arrows). Scale bars: 50 µm.

Gross defects were observed in the ovary of all KO mice. KO ovaries were much smaller and contained reduced follicle numbers (Fig. 6A,B). *In situ* hybridization analysis of ovaries demonstrated that each of the two *STK35* mRNAs and *Stk35os1* are readily detected in somatic cells of WT follicles, with a stronger signal for the short mRNA evident in larger follicles (Fig. 6C). Mating studies with KO females were not performed. These results demonstrate that products of the *STK35* allele are important for normal oogenesis.

### Eye phenotype in *STK35* mutants

**Fig. 7.**
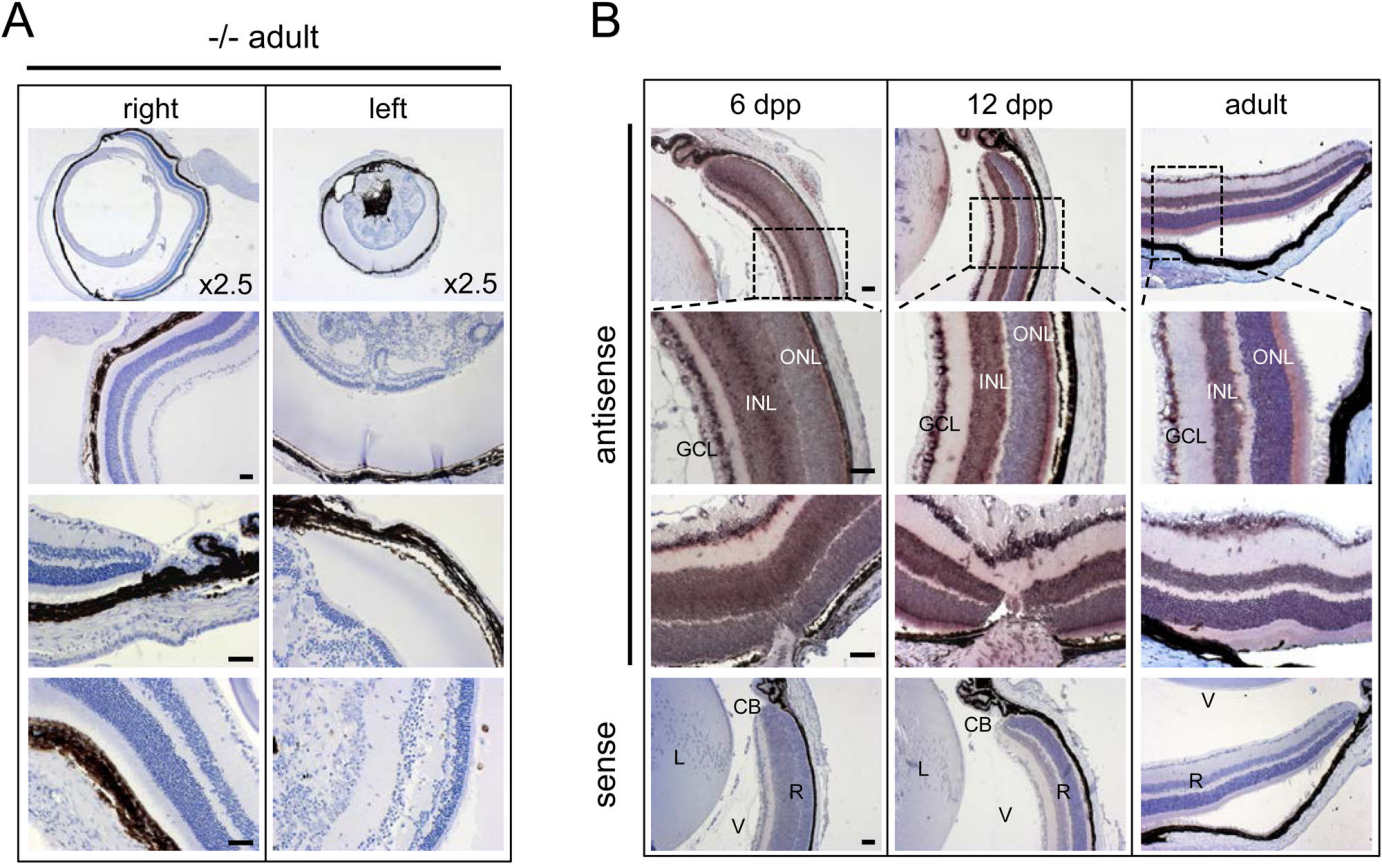
**Eye defects in *STK35* KO mouse.** (A) Histological comparison of right and left eye of *STK35* KO (−/−) adult mouse. (B) *In situ* hybridization of 6 dpp, 12 dpp and adult mouse eye with P2/3 antisense and sense probes. GCL, ganglion cells layer; INL, inner nuclear layer; ONL, Outer nuclear layer; CB, Ciliary body; L, lens; R, retina; V, vitreous. Scale bars: 50 µm.

Abnormal phenotypes were found in some eyes of KO mice (Fig. 7A), ranging from gross deformity (Fig. 7A, left eye) to mild goniodysgenesis or iridocorneal angle deformities. Gross deformities included retinal dysplasia and detachment, lens malformation, iridocorneal adhesions and failure of anterior chamber formation, aniridia (failure or abnormal formation of iris and ciliary body), corneal opacity, microphthalmia and proliferative pigmented masses in the vitreous. *In situ* hybridization analysis using probe P2/3 revealed that *STK35* mRNAs were detected in the ganglion cells layer (GCL) and inner nuclear layer (INL) in 6 dpp and 12 dpp eyes, with the signal intensity reduced in adult eyes (showed P2/3 in Fig. 7B, and P4b data not shown)**.**

## DISCUSSION

This study documents three transcripts, including a lncRNA, that arise from the *STK35* allele with distinct and coordinated expression profiles during mouse spermatogenesis. The finding that the coding mRNAs and lncRNA at this locus are differentially responsive to oxidative stress conditions in mouse germline-derived cells provides evidence these transcripts serve different functions that can reflect changing cell physiological demands.

The *STK35* allele KO mouse model reported here provides unequivocal evidence of its essential contributions to normal embryogenesis, as well as to germline and eye development. The testes of KO mice are significantly smaller and display germline-deficiency, and male KO mice mated with WT females generated litters that are typically smaller than Het-WT matings (3–5 pups compared to 6–10 pups, respectively; Table S2). This indicates that the seminiferous epithelium of the KO mouse can support production of fertile sperm and somatic cells are functionally intact. Sertoli cell marker expression (AMH, SOX9 and CX43) was aberrant in areas lacking germ cells, a presentation that is common in adult seminiferous tubules with spermatogenic disruption. The intriguing observation of intense CX43 staining in areas lacking germ cells suggests this is a direct outcome of *STK35* deficiency, however in the context of a rodent testis model of spermatogenic arrest, overexpression of CX43 was able to support spermatogenic differentiation up to the pachytene stage (Li et al., 2016), in contrast to what is observed in the *STK35* KO testes. Thus, the absence of *STK35* does not appear to directly cause a deficiency in Sertoli cell maturation status that would drive germ cell loss from the seminiferous epithelium (Rajpert-De Meyts et al., 1999; Russell et al., 1990; Sharpe et al., 2003); instead, we hypothesize the deficiency is intrinsic to germ cells arising from the absence of a vital gene product(s) from the *STK35* locus. Initiation of postnatal spermatogenesis involves germ cell migration to the seminiferous epithelium basement membrane (Barakat et al., 2012), while subsequent spermatogenic stages feature progressive movement towards the tubule lumen (Mruk and Cheng, 2004; Siu and Cheng, 2004), hence an inability to migrate could lead to germ cell loss. STK35L1 involvement in migration has been demonstrated in an endothelial cell line (Goyal et al., 2011), and we speculate this could be one important function of a *STK35* gene product in spermatogenesis. The contribution of *STK35* to female fertility remains to be explored.

The *STK35* long mRNA contains the full four exons encoded by the gene NM_183262 and yields the full-length STK35L1 protein when translated from the first start codon, located in exon 1 (described as ‘ATG1’ in Fig. S1). However, western blot analysis revealed the presence of two additional bands at 45 kDa and 50 kDa in the adult mouse testes. Since the short mRNA (AK006778) encodes part of exon 1, with 2nd and 3rd ATGs located in exon 2 (Fig. S1B), these two protein bands may represent alternative forms translated from ‘ATG2’ or ‘ATG3’ of the short mRNA, originally identified as STK35/CLIK1 protein, or its derivative isoforms (Vallenius et al., 2000).

The *Stk35os1* lncRNA overlaps in a head-to-head antisense orientation with the *STK35* protein coding gene. Immediately following exposure of GC-1 cells to oxidative stress, *Stk35os1* significantly increases approximately threefold, while *STK35* transcripts slightly decrease. A recent study of human fibroblast cells demonstrated that the majority of protein coding genes are translationally arrested following oxidative stress, concomitant with the generation of thousands of lncRNAs (Giannakakis et al., 2015). Such information suggests that *Stk35os1* may be part of a plethora of stress-induced lncRNAs that contribute to cellular stress responses in mouse testis. *Stk35os1* is expressed in pachytene spermatocytes and round spermatids, where there is reduced oxygen tension. We thus envisage that synthesis of *Stk35os1* is an essential part of the normal spermatogenic response to the changing local environment as germline cells transition away from the seminiferous tubule base towards the lumen.

Given the close proximity of these transcripts within the genome, we hypothesized that *Stk35os1* could regulate *STK35* expression, however siRNA-mediated knockdown of *Stk35os1*, either in the absence or presence of oxidative stress, did not affect *STK35* transcript levels in a sustained manner. This concurs with studies which show that multiple mRNAs arising from the same genomic locus are not necessarily linked in terms of their function (Goyal et al., 2017).

We propose that coordinated regulation of *STK35* allele transcripts is part of the male germline response to environmental stress that affects cell survival. *STK35* is downregulated in rodent testes following exposure to diesel exhaust particles (Mori et al., 2007). Nuclear-localized importin α2 may be the mechanism by which *STK35* transcription is regulated during spermatogenesis (Hogarth et al., 2007; Miyamoto et al., 2013; Yasuda et al., 2012), and changes in STK35 protein levels may enable cellular adaption processes that ultimately promote cell survival. Levels of the *STK35* transcript are particularly high in adult testis compared to other tissues (Goyal et al., 2009; Vallenius and Makela, 2002). Further experiments to address the regulatory and functional relationships between the coding and non-coding transcripts arising from the *STK35* locus will be relevant to advancing knowledge in fertility, ocular health, and the pathological conditions such as cancers and cardiomyopathy in which *STK35* transcript levels are altered.

## MATERIALS AND METHODS

### Animals and tissue collections

C57BL/6J and Swiss mice were purchased from Monash University Animal Research Platform (MARP). Animals were killed by cervical dislocation [14 days postpartum (dpp) or older] or decapitated (7 dpp). Testes were immediately collected and snap frozen for RNA isolation or fixed in Bouin's solution for 4–5 h before standard embedding and processing for histochemical analyses. The *STK35* KO mouse line was housed in MARP facilities and maintained by heterozygote breeding. In addition, WT, Het and KO males (*n*=4, 17 and 3) were bred with WT females to examine effects on male fertility through assessment of resulting litter size. Experiments were conducted following the National Health and Medical Research Council/Commonwealth Scientific and Industrial Research Organisation/Australian Agricultural Council Code of Practice for the Care and Use of Animals for Experimental Purposes guidelines, and they were approved by the Monash Animal Research Platform Committee on Ethics in Animal Experimentation. Bouin's fixed, paraffin-embedded sections (3–5 μm) were supplied on Superfrost Plus II slides (Menzel-Glaser, Braunschweig, Germany).

### RNA and RNA probe preparation

RNA isolation, production of Digoxygenin (DIG)-labeled riboprobes, northern blot and *in situ* hybridization were performed as previously described (Hogarth et al., 2006). Using primers listed in Table S1, probes P2/3 and P3 were used to amplify a product from the pEGFPC1-STK35 template (Yasuda et al., 2012), and the remaining probes (P1, P1/2, P3/4, P4a, P4b, Pnc1 and Pnc2) were amplified from adult mouse whole testis cDNAs generated by reverse transcriptase using Superscript III reverse transcriptase (Life Technologies) and random hexamer primers (Promega, Madison, USA) according to the enzyme manufacturer's guidelines. Probe positions are shown in Fig. 1A.

### Northern blotting

Northern blots were performed to assess specificity of probe target recognition and to estimate target transcript sizes. Twenty micrograms of total RNA isolated from adult whole mouse testes were separated on 1.2% agarose/formaldehyde gels and transferred to Hybond XL membranes (GE Healthcare Life Sciences). Membranes were blocked with ULTRAhyb™ (Ambion, Austin, USA) at 68°C for 1 h prior to hybridization. DIG-labeled riboprobes (100 ng) were hybridized to membranes overnight at 68°C. Membranes were washed first with 0.1× SSC and 0.1% SDS at 68°C, then in maleic acid buffer (0.1 M Maleic acid, 0.15 M NaCl, pH 7.5, 0.3% Tween). One percent (w/v) blocking reagent (Roche Molecular Biochemicals, Basel, Switzerland) in maleic acid buffer was used to further reduce non-specific binding to membranes and to dilute the anti-DIG-alkaline phosphatase conjugate (anti-DIG-AP; Roche Molecular Biochemicals; 1:10,000). Following a 30 min incubation with anti-DIG-AP antibody, the membranes were washed with the maleic acid buffer and exposed to CDP-Star™ (Roche Molecular Biochemicals) detection reagent for 5 min. Chemiluminescent signal was detected on Hyperfilm™ (Amersham Biosciences, Little Chalfont, UK) for <1 h prior to development.

### *In situ* hybridization

*In situ* hybridization was used to localize *STK35* transcripts in 5 μm sections of Bouin's fixed, paraffin-embedded testis sections from C57BL/6 WT, *STK35* WT or KO mouse testes. Hybridization was performed with 50–200 ng probe per slide at 50°C, 55°C or 60°C, and bound DIG-labeled riboprobe was detected using an anti-DIG antibody conjugated to horseradish peroxidase (Roche Molecular Biochemicals). Antibody binding was visualized as a purple stain by incubating sections in 5-Bromo-4-Chloro-3′-Indolylphosphatase p-Toluidine salt/nitro-blue tetrazolium chloride (BCIP/NBT, Thermo-Scientific). Sections were counterstained with Harris' Haematoxylin (Sigma-Aldrich) to visualize chromatin. Both antisense and sense (negative control) riboprobes were used on each sample, in every experiment, for each set of conditions tested.

### Quantitative real-time PCR

RNA was isolated and gDNA removed from 7, 14, 28 and 60 dpp Swiss mouse testes using TRIzol (Invitrogen) and DNA-free (Ambion). RNA was extracted from cell line samples (GC-1, mouse spermatogonial line) using the RNeasy Mini Kit with on-column DNase-treatment (Qiagen) according to the manufacturer's specifications. For each sample, 500 ng of total RNA was reverse-transcribed in 20 μl reactions with 100 U Superscript III reverse transcriptase (Life Technologies) and random hexamer primers (Promega) according to the enzyme manufacturer's guidelines. Negative control reverse transcription samples lacking Superscript III (-RT) were included for every experiment. Primers used for qRT-PCR are listed in Table S1. Samples were prepared in a final volume of 10 μl using Applied Biosystems *Power* SYBR Green PCR master mix containing 0.5 pmol of each forward and reverse primer (Applied Biosystems, Foster City, USA). PCR was performed on the Applied Biosystems 7900HT Analyzer (MHTP Medical Genomics Facility, Melbourne, Australia) using the following conditions: denaturation at 95°C for 10 min; 40 cycles of amplification at 95°C for 30 s, 62°C for 30 s, and 72°C for 30 s. Each RT+ sample was measured in technical triplicates. Data were analyzed using relative standard curve analysis (SDS 2.3 software), and all values were normalized to the internal control *Rplp0* for the individual sample. Transcript level changes were graphed using GraphPad Prism™ software (GraphPad, San Diego, USA).

### Cell culture, siRNA and hydrogen peroxide treatment

The GC-1 germ cell line (Hofmann et al., 1992) was grown in Dulbecco's modified Eagle's medium (DMEM), containing 10% fetal bovine serum (FBS) at 37°C in 5% CO_2_. GC-1 cells were seeded into 6-well plates at 6×10^4^ cells/well and grown until ∼70% confluent. Cells were transfected with 25 pmol each of the following siRNAs designed by Ambion to target *STK35* lncRNA (NR_029452): Silencer Select Pre-designed siRNA ID: n255157 and n255159 (Invitrogen, no.4390771). The siRNA sense sequences were as follows: n255157: 5′-CCAACAGCCUCGUUGUUAAtt-3′; n255159: 5′-CCUUGGUCAAUUAAAGAGAtt-3′. The following scrambled negative control siRNA was used at 50 pmol: Silencer Select Negative Control #1 (Invitrogen, no.4390844). Transfection with each siRNA was performed using Lipofectamine RNAiMAX (Invitrogen) in accordance with the manufacturer's instructions. After 24 h, GC-1 cells were treated with 1 mM H_2_O_2_ in DMEM lacking FBS for 1 h at 37°C; treatment media was then replaced and cells collected immediately (at 0 h) or at 2 h and 8 h post treatment.

### *STK35* KO mouse line generation

An embryonic stem cell targeting vector was constructed to remove exons 1, 2 and 3 by replacing them with a Flp recombinase target (FRT)-flanked neomycin (Neo) cassette using the Red/ET recombination system (Gene Bridges GmbH, Heidelberg, Germany), as described previously (Wilson et al., 2007). These exons include the start codon of mouse *STK35* and the lncRNA transcript start site in exon 2. Note that the 5′-homology arm included part of exon 1 from −24 to +68 bp. Following electroporation of the targeting construct into Bruce 4 embryonic stem (ES) cell lines, 480 G418-resistant clones were selected for analysis. Thirteen ES clones amplified a product of the expected size from targeted cells by PCR and correctly targeted clones were confirmed by Southern blot analysis at both the 5′ and 3′ ends of the homologous recombination event. Germ line transmissible chimera mice were obtained, and homozygous *STK35* mutation mice (−/−; KOs) were obtained from matings between heterozygous F1 and F2 animals.

Genotyping was performed using 500 ng of genomic DNA for each PCR, amplified by GoTag Flexi DNA polymerase (Promega). Primer sequences are provided in Table S1, with three primers, Ps1, Ps2 and Ps3, used in combination (Fig. 1A). PCR cycle conditions were: 1 cycle at 94°C for 5 min, 45 cycles at 94°C for 30 s, 56°C for 30 s, and 72°C for 50 s and 1 cycle at 72°C for 7 min. Primers Ps1 and Ps2 amplified a 345 bp product from the WT allele, and primers Ps1 and Ps3 amplified a 612 bp product from the targeted flp'd allele (Fig. 1C).

### Immunohistochemistry

Immunohistochemistry was performed as previously (Hogarth et al., 2007) using 5 μm sections of paraffin-embedded Bouin's-fixed mouse testes. Primary antibodies were: Mouse Vasa Homologue (MVH, ab13840-100, 1:500 dilution, Abcam), Anti-Müllerian Hormone (AMH, sc-6886, 1:400, Santa Cruz Biotechnology), Sox9 (sc-20095, 1:100, Santa Cruz Biotechnology) and Connexin 43 (CX43, no.3512, 1:300, Cell Signaling Technology). Primary antibody binding was detected with biotinylated secondary antibodies: rabbit anti-goat (Dako Denmark A/S, Glostrup, Denmark) or goat anti-rabbit (Invitrogen). Signal was amplified using the Vectastain Elite ABC Kit (Vector Laboratories, Burlingame, USA) following the manufacturer's instructions and visualized by incubating sections with hydrogen peroxide-activated 3′,3′-diaminobenzidine tetrahydrochloride (DAB; Sigma-Aldrich). Sections were counterstained with Harris' Hematoxylin (Sigma-Aldrich). Each experiment was performed at least twice on two different samples, with qualitatively identical results observed. Images were obtained using a Zeiss Axioimager microscope running Axio Vision Rel. 4.7 Software (Zeiss, Oberkochen, Germany).

### Western blot

Preparation of mouse testis lysate and western blotting were performed as previously described (Miyamoto et al., 2013; Whiley et al., 2012). STK35 WT, Het or KO testis lysate (30–50 μg per lane) was separated on a 12.5% sodium dodecylsulfate-polyacrylamide gel electrophoresis (SDS-PAGE) gel for Fig. 5C or 15% SDS-PAGE gel for Fig. 5E. Antibodies were: anti-STK35 antibody (Fig. S2, 6G6D2, rat, 1:300 dilution), anti-phospho-Histone H2A.X (Ser139, clone JBW301, Millipore, Mouse, 1:500 dilution), anti-Actin antibody (sc-1616, Santa Cruz Biotechnology, Goat, 1:1000-1:2000), anti-rat IgG-Alkaline Phosphatase (AP) conjugated secondary antibody (A8438, Sigma-Aldrich, 1:4000 dilution), anti-goat IgG-AP antibody (A4187, Sigma-Aldrich, 1:2000 dilution), anti-goat IgG-Alexa Fluor 680 or anti-mouse IRDye800 coupled secondary antibodies (A28088, Thermo Fisher Scientific, or 610132003, Rockland Immunochemicals, Gilbertsville, USA, 1:10,000 dilution).

## Supplementary Material

Supplementary information
