# Supplementary material for: The STK35 locus contributes to normal gametogenesis and encodes a lncRNA responsive to oxidative stress
Source: Biol Open. 2018 Jul 3;7(8):bio032631. doi: 10.1242/bio.032631 (PMC6124569; doi:10.1242/bio.032631)
Supplement: Supplementary information [file biolopen-7-032631-s1.pdf]

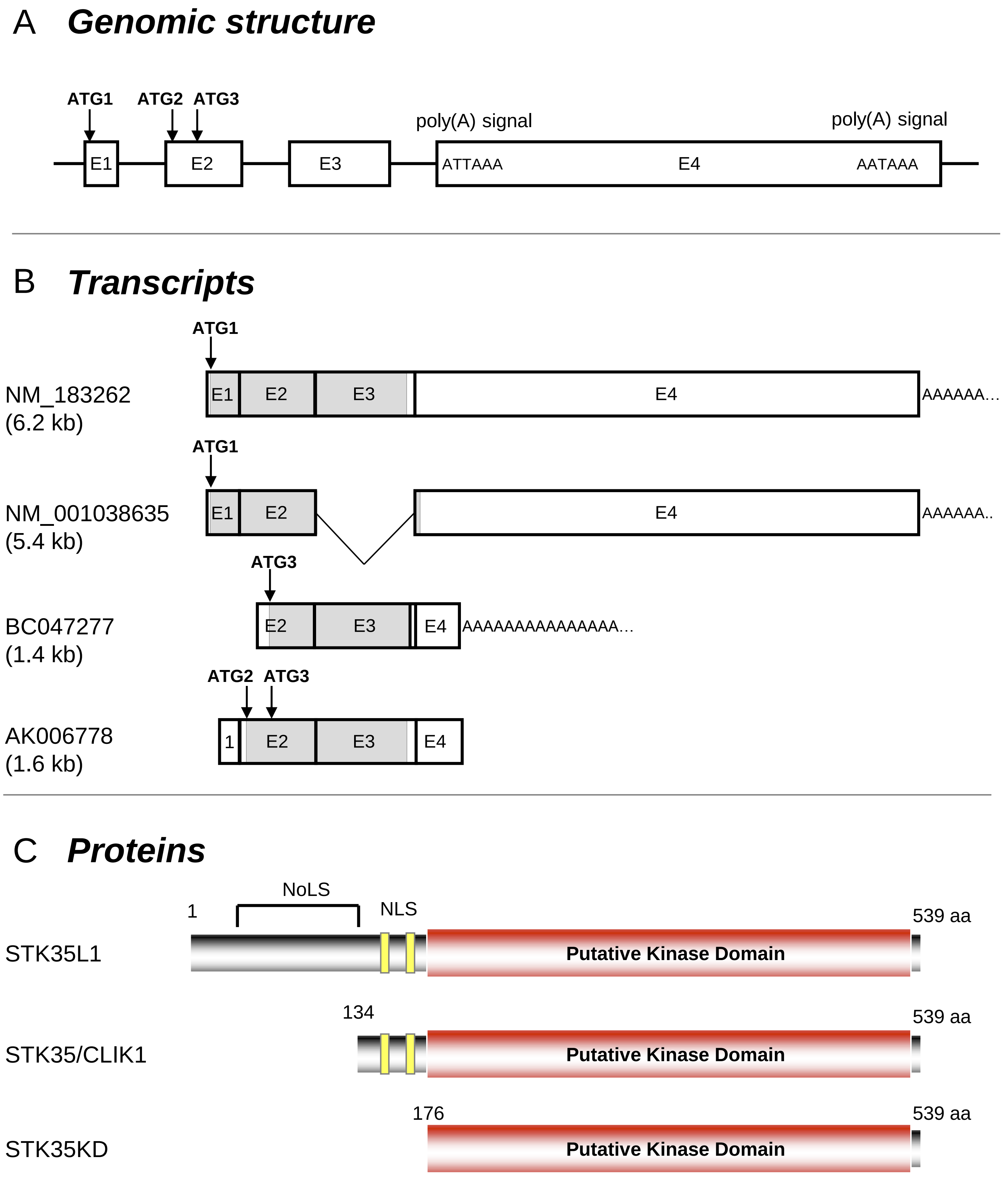

A Genomic structure
ATG1
ATG2
ATG3
poly(A) signal
poly(A) signal
E1
E2
E3
ATTAAA
E4
AATAAA
B Transcripts
ATG1
NM_183262
(6.2 kb)
E1
E2
E3
E4
AAAAAA...
ATG1
NM_001038635
(5.4 kb)
E1
E2
E4
AAAAAA..
ATG3
BC047277
(1.4 kb)
E2
E3
E4
AAAAAAAAAAAAAAA...
ATG2
ATG3
AK006778
(1.6 kb)
1
E2
E3
E4
C Proteins
NoLS
1
NLS
539 aa
STK35L1
Putative Kinase Domain
134
539 aa
STK35/CLIK1
Putative Kinase Domain
176
539 aa
STK35KD
Putative Kinase Domain

Figure S1. Schematic representation of genomic structure, transcripts and protein structure of *Stk35* in mice

A. The Stk35 gene is located on chromosome 2 and its longest isoform consists of 4 exons. Start codons (termed ATG1-3) are located in exons 1 (E1) and 2 (E2). Exon 4 (E4) is designated the three prime untranslated region (3'-UTR) and contains two polyadenylation signals (poly (A) signals) located at either end of the exon.

B. Four transcripts of the *Stk35* gene are identified in NCBI. Three mRNA variants (NM_183262, NM_001038635, BC047277) appear to possess polyadenylation tails and one does not (AK006778). The protein coding region is highlighted in grey, with ATG positions marked. NM_183262 is estimated to be 6.2 kb in length and is comprised of 4 exons. NM_001038635 is estimated to be 5.4 kb long and is presumably a splice variant which lacks exon 3. BC047277 is estimated to be 1.4 kb long and has a partial sequence of exons 2-4 followed by a poly A tail which we presume to be positioned in exon 4. AK006778 is estimated to be 1.6 kb long and is a partial sequence of exons 1-4.

C. Protein structures of STK35. The full-length STK35L1 protein (NP_899085) is translated from ATG1 of NM_183262, and the STK35/CLIK1 and its N-terminus truncated form STK35 kinase domain (KD) are estimated to be translated from ATG2 and ATG3 of AK006778, respectively. A putative nuclear localization signal (NLS, yellow) and an nucleolar localization signal (NoLS, grey) are indicated.

Biology Open (2018): doi:10.1242/bio.032631: Supplementary information

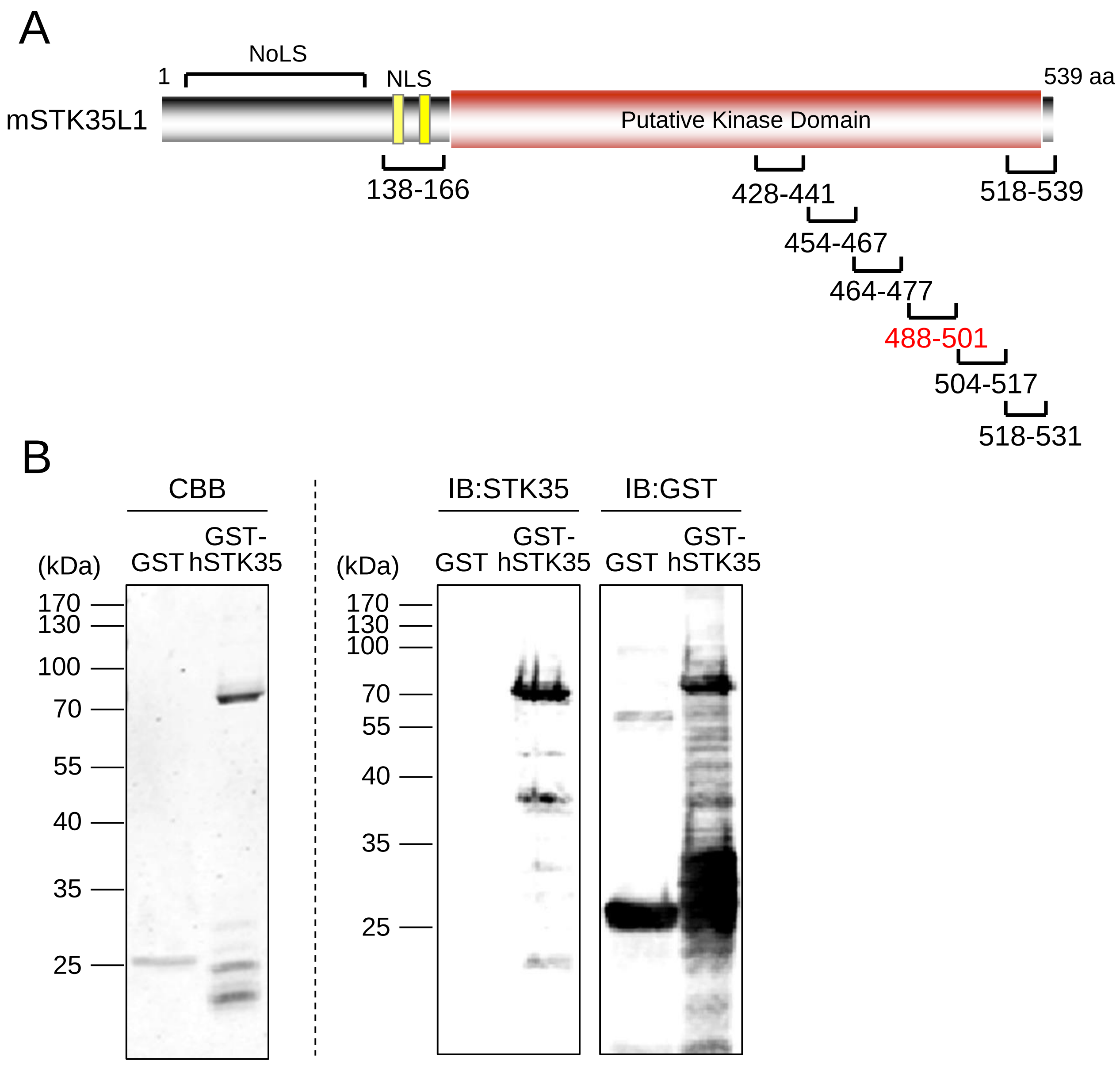

*GST-STK35 is the human STK35 protein.

Biology Open • Supplementary information

Figure S2. Establishment of anti-STK35 antibody

A. Schematic representations of the mouse STK35L1 protein and peptide positions used for immunization. Numbers indicate amino acid. Anti-STK35 rat monoclonal antibodies were generated based on the rat lymph node method established by Sado et al (Kishiro et al., 1995). All animal experiments were performed in accordance with the approved guidelines at the Osaka City University. Immunization and production of the monoclonal antibody were performed as follows: A 10-week-old female WKY/IZM rat (Japan SLC) was injected via the hind footpads with 200 µL of an emulsion containing 167 µg of KLH conjugated peptides and Freund's complete adjuvant. Peptide sequences are as follows; mSTK35L1 (138-166): METGKENGARRGTKSPERKRRSPVQRVLC, mSTK35L1 (518-539): CNPQDRPDAFELETRMDQVTCAA, mSTK35L1 (428-441): CAPEVWEGHYTAKAD, mSTK35L1 (454-467): CERITFIDSETKKEL, mSTK35L1 (464-477): CKKELLGTYIKQGTE, mSTK35L1 (488-501): CNPKMELHIPQKRRT, mSTK35L1 (504-517): CSEGVKQLLKDMLAA, mSTK35L1 (518-531): CNPQDRPDAFELETR. After 3 weeks, the rat was killed, and cells from the lymph nodes of the immunized rat were fused with mouse myeloma Sp2/0-Ag14 cells at a 5:1 ratio in a 50% polyethylene glycol (PEG1500; Merck) solution. The resulting hybridoma cells were plated onto 96-well plates and cultured in HAT selection medium [Hybridoma-SFM medium (Invitrogen), 10% FBS; 1 ng/ml hIL-6 (R&D Systems), 10 mM hypoxanthine, 0.4mM aminopterin, 1.6 mM thymidine]. Six days post-fusion, the hybridoma supernatants were screened by means of an enzyme-linked immunoadsorbent assay (ELISA) against BSA-conjugated peptides. Positive clones were subcloned and rescreened by ELISA and immunoblotting. Finally, two hybridoma clones producing monoclonal antibodies, 5A4A5 and 6G6D2, were selected. Epitope mapping indicated that both recognize the peptide, mSTK35(488-501): CNPKMELHIPQKRRT (show as red). Large-scale *in vitro* production and purification of these antibodies was carried out by culturing clones in serum free medium [Hybridoma-SFM medium containing 1 ng/ml hIL-6] and HiTrap SP ion exchange chromatography (GE Healthcare). The IgG subclass of antibodies was determined using a Rat Immunoglobulin Isotyping ELISA Kit (BD Pharminge), according to the manufacturer instructions. Clones 5A4A5 and 6G6D2 were identified as rat IgG2a(κ).

B. Validation of established STK35 antibodies. Left panel: Coomassie staining of GST or GST-fused human STK35 (hSTK35) recombinant proteins loaded into 12.5% SDS-PAGE gels. The pGEX2T plasmid (GE Healthcare, Tokyo, Japan) was transformed into *Escherichia coli* BL21 and the GST protein was purified as described (Miyamoto and Oka, 2016; Yasuda et al., 2012). The GST-hSTK35 protein was originally produced from baculovirus in Sf9 insect cells (SignalChem, Richmond, BC, Canada). Either 0.25-0.5 ug of the proteins was loaded on the gel, and then the separated proteins in the gel were transferred onto an Immobilon-P membrane (PVDF membrane; Merck Millipore, Darmstadt, Germany). Right panels: Western blot analysis using anti-STK35 antibody (6G6D2) or anti-GST antibody (GE Healthcare).

Biology Open • Supplementary information

REFERENCES

Kishiro, Y., Kagawa, M., Naito, I. and Sado, Y. (1995). A novel method of preparing rat-monoclonal antibody-producing hybridomas by using rat medial iliac lymph node cells. *Cell Struct Funct* **20**, 151-156.

Miyamoto, Y. and Oka, M. (2016). Data on dimer formation between importin α subtypes. *Data Brief.* **7**, 1248-1253

Yasuda, Y., Miyamoto, Y., Yamashiro, T., Asally, M., Masui, A., Wong, C., Loveland, K. L. and Yoneda, Y. (2012). Nuclear retention of importin α coordinates cell fate through changes in gene expression. *EMBO J* **31**, 83-94.

Table S1. Primers

Primer name	5' to 3'
m*Stk35* Genotyping primers	
Ps1	GTGAATCGATTACACCTAAGC
Ps2	CAGCTGAGCACTTTACGTAACC
Ps3	TGCTTTCTCTGAAAACTGTATTCC
Primers for *in situ* hybridization probes and qRT-PCR	
P1 F	ATGGGCCACCAGGAATCTCCGC
P1 R	GCTGTTCCTCTTCCGGGCGAACC
P1/2 F	GGCTCTGCAGACAAGCCCGGCTCG
P1/2 R	TCTGCGGGCTCCGTTCTCTTTCCC
P2/3 F	TCGTGCAGTTTGAGGAGTGCGTCC
P2/3 R	GGATGTTGTCTGGCTTTAGGTCCC
P3 F	CACAGCCAAGGCGGACATCTTTGC
P3 R	TTAAGCAGACACATGTGACCTGGTCC
P3/4 F	CCGAATGGACCAGGTCACATGTGC
P3/4 R	TGCTCTTCAGAAGTCTTGATGG
P4a F	TCCCCTAAGTGCAAGATACATTCC
P4a R	GGGAAGAATGAAAACAGCAGGAGG
P4b F	GCAGGTCTTTGGGTTACCCCTCCC
P4b R	CTCCCCAATACTAGGGGCCCTAGG
Pnc1 F	TTCTGAGGTAGGACCACAATTCAG
Pnc1 R	TCTGTAGGATTGTGGGTACCAGGG
Pnc2 F	ACAAAGATACTCTCTAGCACACTC
Pnc2 R	AACAACCCAGCAACAACAAGG
mRplp0 F	GGACCCGAGAAGACCTCCTT
mRplp0 R	GCACATCACTCAGAATTTCAATGG

Table S2. Breeding strategy for Stk35 mice

Male	Female	Breeding number	Total pups number	Litter size ± SEM
+/+	+/+	4	31	7.8 ± 1.3
+/-	+/+	17	103	6.1 ± 0.5
-/-	+/+	3	12	4.0 ± 0.6

Data are given as mean ± SEM.

Biology Open • Supplementary information
